# Phacoemulsification combined with micropulse cyclodiode laser in glaucoma patients: efficacy and safety

**DOI:** 10.1038/s41433-021-01826-1

**Published:** 2021-11-06

**Authors:** Arij Daas, Thomas Sherman, Lina Danieliute, Saurabh Goyal, Andrew Amon, Ian Rodrigues, Ayesha Karimi, Kin Sheng Lim

**Affiliations:** 1 St Thomas’ Hospital, London, United Kingdom; 2 King’s College London, London, United Kingdom

**Keywords:** Lens diseases, Ocular hypertension

## Abstract

**Objective:**

To evaluate the safety and efficacy of phacoemulsification combined with Micropulse transscleral cyclophotocoagulation (MP-TSCPC) in glaucoma patients.

**Methods:**

This is a retrospective case-note review. The participants were adult patients with diagnoses of glaucoma and cataract who required a further reduction in IOP or a reduction in the number of glaucoma drops. All consecutive patients who underwent cataract surgery (CS) combined with MP-TSCPC laser between October 2018 and July 2019 were included in the study. The effect on visual acuity (VA), intraocular pressure (IOP) and number of anti-glaucoma drops were evaluated at 6 and 12 months in addition to any complications that occurred during any time point of the study.

**Results:**

42 eyes were included in the study. Mean IOP was reduced from 19.5 ± 5.4 mmHg by 22.5% to 15.1 ± 4.6 at 6 months post-operatively and by 19.5% to 15 ± 6.6 mm Hg at 12 months (*p* < 0.001 at both time points). The number of anti-glaucoma medications also reduced significantly from 2.8 ± 1.3 to 1.6 ± 1.2 at 6 months and to 2.2 ± 1.3 at 12 months (*p* < 0.001 at both time points). The success rate was 56% at 6 months and 54% at 12 months. 54.7% of our patients who completed 12 months follow up had an improvement or unchanged vision at the last visits.

**Conclusion:**

This is the first study evaluating the effect of cataract surgery combined with MP-TSCPC in glaucoma patients. We demonstrated that this led to a reduction in IOP and the number of anti-glaucoma medications at 6 and 12-month postoperatively. The majority of patients had either stable or better vision at 12 months follow-up.

## Introduction

Cataract and glaucoma are the leading causes of blindness worldwide [1, 2]. Cataract surgery (CS) is known to reduce IOP by increasing the outflow facility [3]. Micropulse transscleral cyclophotocoagulation (MP-TSCPC) laser is a cyclodestructive process that was first used in 2006; it attempts to reduce IOP by inducing cellular destruction of ciliary processes and associated vasculature, thereby reducing aqueous humour production. However, other theories have been speculated e.g. by Johnstone et al. suggesting that MP-TSCPC might have a similar effect to pilocarpine influencing aqueous humour outflow on ex-vivo study [4]. Previous clinical studies have shown a significant reduction in IOP after using MP-TSCPC in patients with glaucoma [5–8]. Combined procedures have shown a greater effect in reducing IOP in glaucoma patients [9]. The effect of CS combined with MP-TSCPC treatment has never been studied. Therefore, we have performed a retrospective case series analysis of consecutive patients who underwent CS and MP-TSCPC between October 2018 and July 2019 to assess the efficacy of this combined procedure. The qualified success rate was defined as achieving a 20% reduction in IOP with no increase in medications and no further surgery. Secondary outcomes were the change in VA, IOP and the number of antiglaucoma drops used at 6- and 12-months post-surgery as well as any complications.

## Methods

This is a retrospective longitudinal study comparing the change in IOP, VA, and the number of antiglaucoma agents used by consecutive patients who underwent CS with MP-TSCPC at St. Thomas’ Hospital between October 2018 and July 2019. The data are extracted from the electronic patient records and paper notes. For the purpose of this study, baseline up to 12 months postoperative follow up data were analysed. Phacoemulsification combined with MP-TSCPC was offered to optimise IOP in mild, moderate and advanced glaucoma patients who were undergoing cataract surgeries. Patients with normal tension glaucoma (NTG), open angle (OAG) and primary angle closure glaucoma (PACG) were included in the study, we have excluded patients with neovascular glaucoma and uveitic glaucoma from our study. Informed consent was obtained from patients undergoing surgery. We conducted the study within an ethical framework, as outlined in the Declaration of Helsinki.

### Surgical technique

Surgery was performed by experienced surgeons, two consultants (KSL & SG) and one glaucoma fellow. Peribulbar anaesthesia was performed prior to the combined procedures followed by Micropulse cyclodiode laser using the following parameters: 2 W delivered over 160 s with a sweeping motion avoiding the 3 and 9 o’clock positions. This was followed by uncomplicated cataract surgery performed with a standard phacoemulsification technique. All patients received intra-cameral cefuroxime (0.1 mg/0.1 mL) and dexamethasone (0.2 mL) at the end of the surgery. Topical and oral glaucoma medications were stopped on the operated eye at the time of surgery. Patients were given chloramphenicol eye drops QDS for 1 week and dexamethasone preservative-free eye drops 2 hourly for 1 week on a tapering regime over the next 6 weeks.

### Follow up

All patients had follow-up at 1 week, 3, 6 and 12 months. If they had any complications, the frequency of the follow up was adjusted according to clinical need.

### Data analysis

Data were recorded on Microsoft Excel 2016 and statistical analysis was performed using SPSS 25.0; SPSS, Chicago, IL. Means with standard deviations (SD) were calculated for IOPs and means with 95% confidence intervals were calculated where appropriate at all time points. The Chi-square goodness of fit test showed that IOP followed a normal distribution at all time points; therefore, the paired Student’s *t* test was used to compare baseline and postoperative mean IOP. One-way ANOVA was performed to compare the change in outcome measures during baseline and follow-up periods. A *p* < 0.05 was considered statistically significant.

## Results

**Fig. 1:**
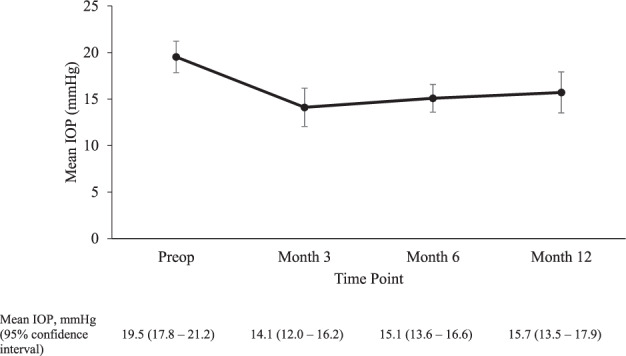
Change in IOP over time. Mean IOP at pre- and post- combined MP-TSCPC and cataract surgery at Month 3, Month 6 and Month 12, shown with 95% confidence intervals.

**Fig. 2:**
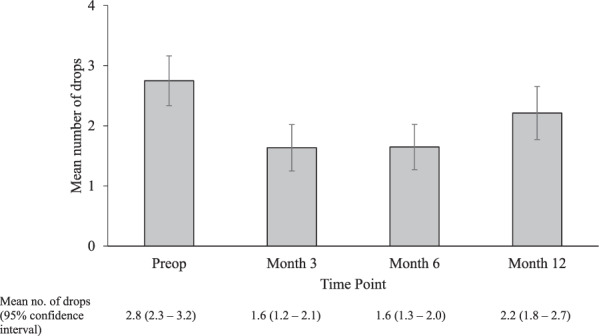
Change in number of glaucoma eye drops over time. Mean number of drops at pre- and post- combined MP-TSCPC and cataract surgery at Month 3, Month 6 and Month 12, shown with 95% confidence intervals.

42 eyes were included in the study with 34 reaching 12 months follow up. Baseline demographics and clinical characteristics of the patients included in the study are shown in (Table 1) The mean baseline IOP was 19.5 ± 5.4 mmHg and ranged from 9 to 40 mmHg. The mean IOP at 6 and 12 months was 15.1 ± 4.6 and 15.7 ± 6.6 mmHg, respectively (Fig. 1). This was a statistically significant reduction at both follow-up timepoints, with a *p* < 0.001. 22.5% and 19.5% IOP reduction was achieved at 6- and 12-months follow-up, respectively. The mean number of antiglaucoma agents used at baseline was 2.8 ± 1.3 only one patient was on oral Acetazolamide. This reduced to 1.6 ± 1.2 and 2.2 ± 1.3 at 6- and 12-months and was statistically significant at both time points (Fig. 2) none of the patients required oral Acetazolamide at 12 months follow up. The overall success rate was 56% at 6 months and 54% at 12 months.

**Table 1:** Patient demographics.

No. of surgeries	42
Age, years	
Mean (Range)	74.9 (53–89)
Sex, *n* (%)	
Male	54.70%
Female	45.20%
Ethnicity, *n* (%)	
Afro-Caribbean	40.40%
White	45.20%
Asian	11.9
Mixed (white & Afro-Caribbean)	2.30%
Pre-op IOP, mmHg	
Mean (95% confidence interval)	19.5 (17.8–21.2)
Range	9–40
No. of IOP lowering medications pre-op	
Mean (95% confidence interval)	2.8 (2.3–3.2)
Range	0–4
Diagnoses, *n* (%)	
POAG	34 (81.0)
PACG	4 (9.5)
NTG	2 (4.8)
PDG	1 (2.4)
OHT	1 (2.4)
Glaucoma severity, *n* (%)	
Mild	8 (19.0)
Moderate	18 (42.9)
Severe	16 (38.1)

*POAG* primary open angle glaucoma, *PACG* primary angle closure glaucoma, *NTG* normal tension glaucoma, *PDG* pigment dispersion glaucoma, *OHT* ocular hypertension.

**Fig. 3:**
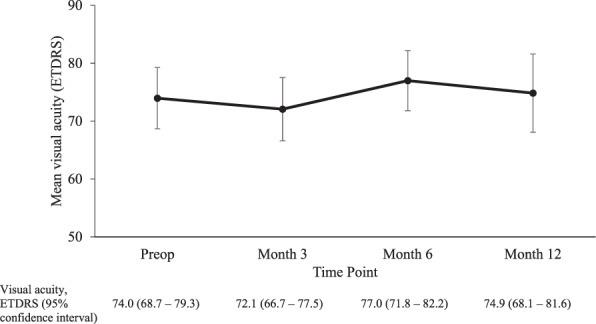
Change in visual acuity over time. Mean visual acuity at pre- and post- combined MP-TSCPC and cataract surgery at Month 3, Month 6 and Month 12, shown with 95% confidence intervals.

There was no statistically significant improvement on visual acuity, from ETDRS 74 ± 17 at baseline to 75 ± 20 at 12 months (Fig. 3). 42.8% of our patients who completed 12 months follow up had an improvement in their visual acuity by 1 line or more, 11.9% had unchanged vision and 26.1% (11 patients) had worsening in their vision.

Complications included cystoid macular oedema (CMO), persistent anterior uveitis, persistent mydriasis, further surgery for glaucoma and visual acuity loss of >2 Snellen lines (Table 2). Five patients had cystoid macular oedema at the 3-month timepoint: Two of these cases improved by 6 months through drops and observation and one improved by 12 months after a subconjunctival triamcinolone injection. One patient that developed CMO had a previous branch retinal vein occlusion in the eye. Unfortunately, one patient had newly developed CMO that started 1-week post-surgery, and this persisted beyond 12 months. Ten patients had anterior uveitis and two patients required anterior chamber (AC) tissue plasminogen activator (tPA) injection within the first three months post-op. One patient had persistent anterior chamber inflammation post-op twelve months whereas another case developed new AC activity at 12 months. Further glaucoma filtration surgery was required for five patients at different timepoints, comprising of four trabeculectomies and one Baerveldt tube. Of these patients, two had primary open angle, two had primary angle closure and one patient had pseudoexfoliation glaucoma. Though six cases had a visual acuity loss of >2 Snellen lines at 3 months compared to preoperatively, five additional patients had a new visual acuity loss recorded at 12 months due to one case requiring a Trabeculectomy, one Baerveldt, one persistent anterior uveitis, one developed posterior capsular opacification and the reason for one was unknown.

**Table 2:** Complications occurring at different timepoints following micropulse transscleral cyclophotocoagulation combined with cataract surgery.

Complication	3 months	6 months	12 months
Cystoid macular oedema	5	0	1
Persistent anterior uveitis	10	1	2
Persistent mydriasis	2	1	0
Further surgery for glaucoma	1	3	1
VA loss of >2 Snellen lines	6	0	5

## Discussion

This is the first study to evaluate the effect of CS combined with MP-TSCPC in glaucoma patients. We demonstrated that this was effective in reducing IOP and the need for anti-glaucoma drops at all timepoints.

Previous studies have found the decrease in IOP to be between 1.5 and 9.5 mmHg after phacoemulsification [10–14]. The drop in IOP by 9.7% can persist up to 5 years follow-up in 76% of the glaucoma patients [13]. The reduction in IOP after MP-TSCPC however is reported to be 20–60% [5–7, 15]. We have demonstrated that IOP lowered by 22.5% at 6 months and 19.5% at 12 months follow-up when cataract surgery was combined with MP-TSCPC.Though the IOP appeared to increase up to the month 12 timepoint, this was not statistically significant (*p*: 0.19).

Although the level of IOP lowering appeared to be similar to some studies following CS alone, our result compared well with another study from our unit [3] where CS alone achieved a 12% reduction of IOP at 3 months. This shows there may be confounding factors in the difference in IOP reduction compared to other studies, including patient cohort, surgical technique, drop choice, etc. The reduction in IOP can be explained by the increase in tonographic outflow facility after CS [3] and also by the decrease in aqueous humour production, which is the most widely held view regarding the mechanism of IOP reduction post MP-TSCPC. The change in anti-glaucoma drops was consistent with Tan et al.’s findings [6].

The change in IOP at 6 and 12 months after receiving MP-TSCPC alone have been inconsistent in previous studies. In the report by Zaarour et al. [15], it was found that the mean IOP reduced from month 6–12 and this may have been due to IOP-raising postoperative inflammation, which improved by 12 months as compared to 6 months. Others have reported unchanged IOP with time [16]. Data on this are conflicting, and all are based on MP-TSCPC alone, rather than combined with cataract surgery as in our study. We found that IOPs after surgery overall started to rise by 12 months compared to 6 months, which is the general trend following majority of glaucoma and cataract surgeries.

Our study demonstrated that 42.8% of our patients who completed 12 months follow up had an improvement in their visual acuity by 1 line or more, 11.9% had unchanged vision and 26.1% (11 patients) had worsening in their vision. We explored the patients who had worsening in vision and found: 2 patients had further glaucoma filtrating surgeries, 2 patients had CMO, 1 patient had persistent inflammation, and 1 patient had posterior capsular opacification at 12 months follow up.

The incidence of CMO has been reported to be between 1% and 30% [17, 18] after non-complicated cataract surgery and 5% after only MP-TSCPC [19]. Two patients developed CMO which was persistent at the 1 year follow up. One of these patients had a previously established branch retinal vein occlusion in the operated eye as a confounding factor, and the other had severe anterior chamber inflammation and presented with CMO 1 week postoperatively. We found that severe inflammation in the early days post-surgery may be an indication towards poor prognosis long term. Therefore, aggressive anti-inflammatory treatment should be considered early. Perez et al. reported neurotrophic keratitis in two patients after receiving MP-TSCPC [20]. However, our study did not show any patients with this complication.

Our study demonstrates promising results, longer follow-up should be sought to assess the influence on IOP reduction beyond 12 months. A randomised trial could be considered comparing phacoemulsification alone versus phacoemulsification with MP-TSCPC in one type of glaucoma, such as OAG, to truly understand the many factors affecting IOP reduction.

### Summary

#### What was known before

- Cataract surgery is known to reduce the intra-ocular pressure by increasing the outflow facility.
- Micropulse cyclodiode laser is also known to reduce the intra-ocular pressure.
- The effect of cataract surgery combined with Micropulse cyclodiode laser has never been studies.

#### What this study adds

- This is the first study to evaluate the effect of cataract surgery combined with micropulse cyclodiode laser in glaucoma patients.
- We demonstrated that this was effective in reducing IOP and the need for anti-glaucoma drops at all timepoints.
